# Therapeutic Effect of *Ecklonia cava* Extract in Letrozole-Induced Polycystic Ovary Syndrome Rats

**DOI:** 10.3389/fphar.2018.01325

**Published:** 2018-11-19

**Authors:** Hyun Yang, Seung Yeon Lee, Sang R. Lee, Bo-Jeong Pyun, Hye Jin Kim, Young Ho Lee, Sun Woo Kwon, Dong Ho Suh, Choong Hwan Lee, Eui-Ju Hong, Hye Won Lee

**Affiliations:** ^1^Herbal Medicine Research Division, Korea Institute of Oriental Medicine, Daejeon, South Korea; ^2^College of Veterinary Medicine, Chungnam National University, Daejeon, South Korea; ^3^Department of Bioscience and Biotechnology, Konkuk University, Seoul, South Korea

**Keywords:** *Ecklonia cava*, polycystic ovary syndrome, follicle stimulating hormone, luteinizing hormone, androgen, testosterone, dehydroepiandrosterone

## Abstract

Polycystic ovary syndrome (PCOS) is an endocrinal disorder that afflicts mainly women of childbearing age. The symptoms of PCOS are irregular menstrual cycles, weight gain, subfertility and infertility. However, because the etiology is unclear, management and treatment methods for PCOS are not well established. Recently, natural substances have been used for PCOS therapy. *Ecklonia cava* (*E. cava*) is a well-known natural substance that attenuates the effects of inflammation, allergies, and cancer. In this study, we investigated the effects of *E. cava* extract in rats with PCOS. When rats with letrozole-induced PCOS were exposed to the *E. cava* extract, the regular estrus cycle was restored, similar to that in placebo rats. Hormone levels, including the levels of testosterone, estrogen, luteinizing hormone (LH), follicle stimulating hormone (FSH), and anti-Müllerian hormone (AMH), were restored to their normal states. Histological analysis revealed that the polycystic ovary symptoms were significantly decreased in the *E. cava*-treated rats and were comparable to those of normal ovaries. At the transcriptional and translational levels, *Ar*, and *Esr2* levels were markedly increased in the *E. cava*-treated rats with PCOS compared with the rats with letrozole-induced PCOS. These results suggest that the *E. cava* extract inhibits the symptoms of PCOS by restoring imbalanced hormonal levels and irregular ovarian cycles in letrozole-induced female rats.

## Introduction

Polycystic ovary syndrome (PCOS) is a common endocrinal disorder that afflicts women of childbearing age. The common symptoms of PCOS include irregular menstrual cycles, weight gain, subfertility, and infertility (Sirmans and Pate, 2013). The symptoms also include lack of ovulation, high androgen levels and high numbers of ovarian cysts. Two of the above three parameters are used to diagnose PCOS in women (Rosenfield and Ehrmann, 2016). Approximately three-quarters of PCOS patients have high androgen levels, and this hyperandrogenism is thought to be genetically acquired and an effect of the environment on the hypothalamic-pituitary-ovarian axis (Codner and Escobar-Morreale, 2007).

A deficiency in aromatase activity is one of the intraovarian disturbances in steroidogenesis that is thought to trigger ovarian failure, such as in PCOS. Because aromatase catalyzes the rate-determining step during the biosynthesis of estrogens from androgens, decreased activity of this enzyme could be expected to result in hormonal imbalance, circulating hyperandrogenism and intraovarian androgen excess, resulting in polycystic ovaries (Kafali et al., 2004; Caldwell et al., 2014). Letrozole has been administered to female rats that were used as animal models of PCOS (Caldwell et al., 2014). In PCOS models, follicular atresia and abnormal follicular development have also been observed in polycystic ovaries (Choi et al., 2005). Aromatase, encoded by the *Cyp19* gene, is a member of the cytochrome P450 family (Bulun et al., 2003) and is a rate-limiting enzyme that catalyzes the conversion of androgen to estrogens during steroidogenesis (Mills et al., 2014). In the ovary, estradiol is generated by the conversion of C19 androgens derived from theca cells under the influence of aromatase produced by granulosa cells (Lazaros et al., 2013). In addition to high testosterone concentrations, women with PCOS exhibit decreased expression of the estrogen receptor β in the granulosa cell layer of cystic follicles; this effect has also been observed in animals treated with letrozole compared to normal animals.

*Ecklonia cava* is a brown alga that found on Jeju island, Korea, and in Japan (Kim et al., 2006). *E. cava* is widely used as a functional food, supplement and ingredient for animal feed (Lee et al., 2010). Recently, *E. cava* received attention due to its biological activities: attenuation of inflammation, antiallergenic activity, antidiabetic activity, antioxidant activity and anticancer activity (Venkatesan et al., 2016; Hwang et al., 2017; Kim and Nam, 2017). *E. cava* extracts contain polyphenolic compounds that are referred to as phlorotannins, including eckol, 6,6′-bieckol, 8,8′-dieckol, dieckol, phlorofucofuroeckol, phloroglucinol, and triphlorethol-A (Kang et al., 2005). Dieckol is one of the major polyphenolic compounds present in *E. cava*. Among the numerous therapies available for management of PCOS and induction of ovulation, *E. cava* is a seaweed that is known to be one of the richest sources of phloroglucinols, which are potent and highly effective antioxidants, antibacterials and anti-inflammatory agents (Rocha et al., 1995; Weng et al., 2004; Goswami et al., 2016). Other beneficial effects include anticancer properties. Topical application of *E. cava* is known to enhance hair growth; this is a novel application that also leads to inhibition of androgen-induced hair loss (Kang et al., 2012).

Although not described for PCOS therapy, natural substances are becoming increasingly common and are replacing known medications for treatment of PCOS symptoms. Given our interest in potential applications of *E. cava* extract as an alternative medicine and as a treatment for PCOS, we evaluated the reproductive, endocrine and metabolic features during assessment of the effectiveness of this extract in a letrozole-induced rat model of PCOS.

## Materials and Methods

### Preparation of *E. cava* Extract

*Ecklonia cava*, collected from Jeju island of South Korea, was purchased from a comprehensive herbal medicinal shopping mall at Jungwoo-Dang (South Korea). Briefly, dried *E. cava* (5 kg) was extracted with 75 L of water for 3 h with refluxing at 101 ± 1°C and then filtered. To obtain *E. cava* powder, the extract was concentrated and lyophilized in a freeze-dryer. The final yield of *E. cava* extract was 11.46% w/w (573.01 g). The plants were deposited in the Herbal Medicine Research Division of Korea Institute of Oriental Medicine (KIOM) in Daejeon, Korea (voucher specimen KIOM H 160150).

### Quantitative Analysis of *E. cava* Extract

The five reference compounds present in *E. cava*, namely, eckol, dieckol, 6,6′-dieckol, 8,8′-dieckol and phloroglucinol (200 μg/mL), were dissolved in 80% methanol. To quantity the 5 metabolites of *E. cava*, each analyte was studied in multiple reaction monitoring (MRM) mode. The dried *E. cava* extracts (5 mg) were reconstituted in 1 mL of 80% methanol and filtered using a 0.45-μm syringe filter (13 mm, Ann Arbor, MI, United States). All samples aliquots (5 μL) were injected into a liquid chromatography (LC)-mass spectrometry (MS) system. LC-MS/MS analysis was performed on a Nexera2 LC system (Shimadzu Corp., Kyoto, Japan) combined with a triple quadrupole MS (LC-MS 8040, Shimadzu) equipped with an electrospray source. Separation was performed on a Kinetex C18 column (100 mm × 2.1 mm, 2.6 μm, Phenomenex, Torrance, CA, United States). The eluent consisted of water containing 0.1% formic acid (solvent A) and acetonitrile containing 0.1% formic acid (solvent B), and the flow rate used was 300 μL/min. The following gradient was used: solvent B was maintained at 5% for 1 min, linearly increased from 5% to 100% over 9 min, and then decreased to 5% over 1 min. The MS was operated under the following conditions: capillary voltage, -3000 V; capillary temperature, 350°C; vaporizer temperature, 300°C; sheath gas, 3 L/min; ion sweep gas, 2.0 arb; aux gas, 10 arb, and drying gas, 8 L/min. For quantification, the following MRM transitions were used in this study: 371 → 263 for eckol, 741 ? 261 for dieckol, 741 → 477 for 6,6′-bieckol, 741 → 723 for 8,8′-bieckol, and 125 → 57 for phloroglucinol. The LC-MS MRM chromatograms of the 5 metabolites are summarized in Supplementary Figure S1.

### Cell Culture and Determination of Cell Viability

Human adrenal NCI-H295R cells were obtained from American Type Culture Collection (ATCC-LGC Standards GmbH, Wesel, Germany). Cells were cultured under standard conditions in Dulbecco’s modified Eagle’s/Ham’s F-12 medium (DMEM/F12; Gibco, Life Technologies Europe BV, Bleiswijk, Netherlands) supplemented with 2.5% Nu-serum (BD Biosciences, Breda, Netherlands), 1% ITS (insulin/transferrin/selenium; BD Biosciences), 1% penicillin/streptomycin (Pen/Strep; Gibco, Life Technologies). Other biochemical reagents, including metformin and pioglitazone, were obtained from Sigma-Aldrich (St. Louis, MO, United States), unless otherwise specified. The NCI-H295R cells were seeded into 96-well plates at a density of 1 × 10^3^ cells/well and incubated in serum-free conditions prior to treatment with FOR, *E. cava* water extract, metformin, or pioglitazone. Cell viability was evaluated by the 3-(4,5-dimethylthiazol-2-yl)-2,5-diphenyltetrazolium bromide (MTT) assay. After treatment, MTT solutions (0.5 mg/mL) were added to each well, and the plates were incubated for 4 h at 37°C. The supernatant was removed, and the formazan product obtained was dissolved in 100 μL of dimethyl sulfoxide (DMSO) with stirring for 15 min on a shaker. The absorbance was measured with a microtiter plate reader (BIO-TEK, Synergy HT, Winooski, VT, United States) at 570 nm. The percentage of viable cells in each treatment group was determined by the experimental optical density (OD) value of the control.

Human breast cancer cells (MCF-7) were maintained in culture flasks in full medium, consisting of DMEM supplemented with 5% heat-inactive fetal bovine serum. The cells were maintained in a humidified incubator at 37°C and with 5% CO_2_. After 24 h, the cells were washed with PBS, and the medium was replaced with estrogen-free medium (phenol red-free DMEM with 2% charcoal:dextran-stripped bovine serum) for 48 h. MCF-7 cells were counted using an improved Neubauer counting chamber. Separately treated MCF-7 cells were used for RNA analysis.

### Dehydroepiandrosterone (DHEA) Measurements

Dehydroepiandrosterone concentrations were measured using a competitive enzyme-linked immunosorbent assay (ELISA) kit (Enzo Life Sciences, Sigford Road, Exeter, United Kingdom) following the manufacturer’s instructions. The NCI-H295R cells were plated into 96-well plates at 1 × 10^3^ cells/well and incubated in serum-free conditions before exposure to forskolin (FOR) (10 μM) in the presence or absence of *E. cava* water extract, metformin, or pioglitazone for 24 h. The levels of DHEA released into the media were determined in triplicate against standards prepared in medium using the ELISA kit. The results were normalized to the controls.

### Animals

Sprague Dawley rats were obtained from Orient Bio Inc. (Seongnam, South Korea) and were housed in a pathogen-free facility at Chungnam National University under a standard 12 h light:12 h dark cycle and fed standard chow with water provided *ad libitum*. Rats were acclimated to laboratory conditions for 1 week. All rat experiments were performed in accordance with the recommendations of the Chungnam University Facility Animal Care Committee (CNU-00606), following the guidelines of the Association for Assessment and Accreditation of Laboratory Animal Care International. For PCOS experiments, letrozole pellets (IRA; Innovative Research of America, OH, United States; 0.1 mg/kg/day) were administered to 6-week-old female rats in an animal anesthesia system (Vetequip^®^, Abesko, Gyeonggi, Korea) using 2% isoflurane. After 2 weeks, the rats were administered *E. cava* extract per os (P.O.) every day for 2 weeks.

### Serum Hormone Analysis

Blood samples were collected directly from the inferior vena cava using a 1-mL syringe at the end of the experiment. Serum was obtained by centrifugation at 2,000 × g for 10 min and stored at -70°C until use. Serum luteinizing hormone (LH), follicle stimulating hormone (FSH), and anti-Müllerian hormone (AMH) levels were measured using ELISA kits (Cusabio Biotech, Wuhan, China). Serum testosterone levels were measured using a Testosterone ELISA Kit (Abcam, Cambridge, United Kingdom). The 17β-estradiol (E2) levels were measured using an Estradiol (Rat) ELISA Kit (BioVision, United States). All hormone levels were measured according to the manufacturer’s instructions.

### Reverse Transcription and Real-Time PCR

Total RNA was extracted using TRI reagent (Molecular Research Center, Cincinnati, OH, United States) following the manufacturer’s instructions. cDNA was synthesized from 1 μg of total RNA using a Thermo Scientific RevertAid First Strand cDNA Synthesis Kit (Thermo Corporation, MA, United States). The cDNA was amplified by RT-PCR using AmpliTaq Gold DNA polymerase and quantitative real-time PCR and by using Premix ExTaq (TaKaRa, Shiga, Japan) with SYBR Green I (Molecular Probes, Eugene, OR, United States) in a Step One Plus system (Applied Biosystems). The primers were synthesized by Macrogen Inc. (Seoul, Korea). *Actin* expression was used as a control. The primers used for RT-PCR are shown in Table 1. All experiments were performed in triplicate, and mRNA values were calculated based on the cycle threshold and monitored to obtain an amplification curve.

**Table 1:** Sequence identification and primers used for RT-PCR analysis.

No.	Gene	Forward Primer (5′ to 3′)	Reverse Primer (5′ to 3′)
1	*Kitl*	GGTAGCCAGGAGTTTGTTCT	TTGTGTGGCATAAGGGCT
2	*Bmp*	GATATTGAGTCTCAGCCCGA	AACATGCGGTTGCCTGTA
3	*Has2*	TCAATGGGGGTTGGTTTCTT	GGGAAAAGTGCATAAGCCA
4	*Fshr*	CTCATCAAGCGACACCAAGA	GGAAAGGATTGGCACAAGAA
5	*Fshb*	GGACCCAGCTAGACCAAACA	GTCCCAGGCCTCTTACAGTG
6	*Lhr*	ACACTGCCCTCCAAAGAAAA	CCTCAAAGATGGCGGAATAA
7	*Ar*	CTGGGAAGGGTCTACCCAC	GGTGCTATGTTAGCGGCCTC
8	*Esr2*	AGTAGCCGGAAGCTGACACA	CATGCTGAGCAGATGTTCCA
9	*Cyp11a1*	TCCTCAAAGCCAGCATCA	ATCTCGACCCATGGCAAA
10	*Cyp19a1*	ATGTTCTTGGAAATGCTGAACCC	AGGACCTGGTATTGAAGACGAG
11	*pS2*	CCCCGTGAAAGACAGAATTGT	GGTGTCGTCGAAACAGCAG
12	*Gapdh*	GCTGAGTATGTCGTGGAGTC	TTGGTGGTGCAGGATGCATT
13	*Actin*	TACGTCGCCCTGGATTTT	ATGAAAGAGGGCTGGAAGAG

### Histological Analysis and Immunohistochemistry

Ovaries were fixed in 10% buffered formalin for 48 h and subsequently embedded in paraffin. Paraffin-embedded tissue sections were dewaxed, rehydrated and stained with hematoxylin and eosin (H&E; Sigma-Aldrich). The stained slides were examined using a VM600 digital slide scanning system (Motic, CA, United States). To break protein cross-links, the tissue sections were incubated with 0.1 M citrate buffer (pH 6.0) at 95–100°C for 1 h. After blocking with 3% BSA, slides were incubated with primary antibodies at 4°C overnight. Then, the slides were washed and incubated simultaneously with the corresponding Alexa-Fluor-conjugated secondary antibodies (Life Technologies) diluted in TBS with 1% BSA at room temperature for 1 h. After washing, the slides were mounted in ProLong Gold antifade reagent and examined using a DMi8 microscope (Leica Microsystems, Wetzlar, Germany).

### Statistical Analysis

The results obtained for the NCI-H295R cell proliferation, DHEA production and follicular genetic marker assays are presented (± SD). The other results are reported as the mean ± SEM. Differences between means were obtained by Student’s *t*-test, and ANOVA (Tukey-Kramer method) was performed using Graph Pad software (GraphPad Inc., San Diego, CA, United States).

## Results

### Quantitative Analysis of Major Compounds in *E. cava* Extract

The *E. cava* extract and five major reference compounds (eckol, dieckol, 6,6′-dieckol, 8,8′-dieckol, and phloroglucinol) were subjected to UHPLC-Triple-Q-MS/MS analysis. The MRM mode of triple-quadrupole MS was used to achieve high-sensitivity quantification of the specific molecules. The 5 metabolites were quantified using the LC-MS MRM method, and the measured concentrations are listed in Supplementary Table S1. The quantification data for the 5 metabolites are as follows: dieckol (4.6478 μg/mg of sample) ≥ 6,6′-bieckol (4.6444 μg/mg of sample) > 8,8′-bieckol (1.1841 μg/mg of sample) ≥ eckol (1.01246 μg/mg of sample) > phloroglucinol (0.185 μg/mg of sample). Analysis of the contents of the *E. cava* extracts revealed maximum concentrations of dieckol and 6,6′-bieckol compared to other standards.

### Effect of *E. cava* Extract on Human Adrenal Cell Viability

**FIGURE 1:**
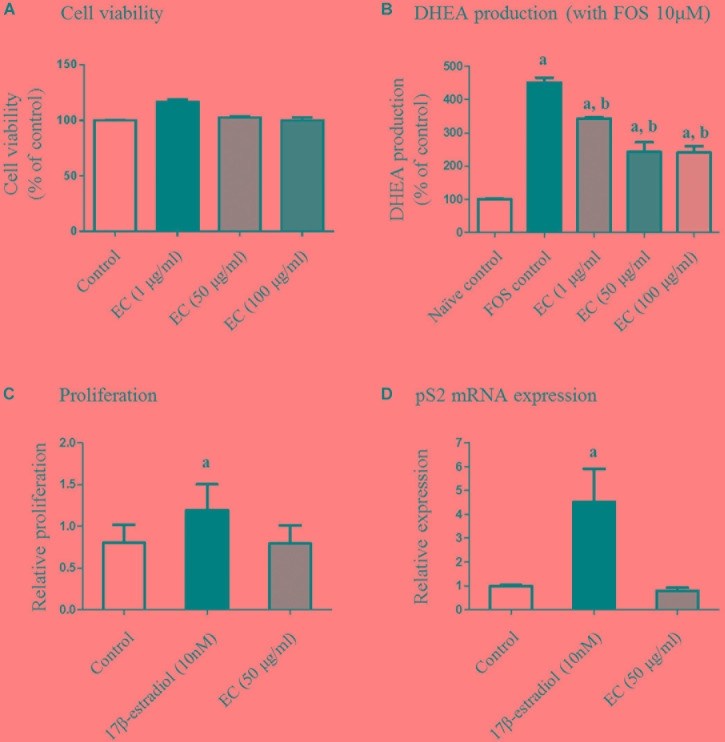
Effect of *E. cava* extract on human adrenal NCI-H295R cells **(A,B)** and MCF-7 cells **(C,D)**. **(A)** NCI-H295R cells were incubated in the presence of the indicated concentrations of *E. cava* extract for 24 h. Cell viability was determined using the 3-(4,5-dimethylthiazol-2-yl)-2,5-diphenyltetrazolium bromide (MTT) assay and measurement of absorbance at 540 nm. **(B)** Cells were pretreated with *E. cava* extract (1, 50, and 100 μg/mL) for 30 min prior to exposure to 10 μM forskolin (Sigma-Aldrich) for 24 h. DHEA production was measured in cell culture medium by a competitive enzyme-linked immunosorbent assay following the manufacturer’s recommendations. Data are representative of three independent experiments and are expressed as the mean ± SD; ^a^*p* < 0.05 vs. control; ^b^*p* < 0.05 vs. forskolin. **(C)** MCF-7 cell proliferation and **(D)** *pS2* gene expression were examined after treatment with estrogen and *E. cava* extract in estrogen-free medium (phenol red-free DMEM with 2% charcoal:dextran-stripped bovine serum) for 48 h. Data are representative of three independent experiments and expressed as the mean ± SD; ^a^*p* < 0.05 vs. control. EC, *E. cava* extract; PCOS, polycystic ovary syndrome.

To determine non-toxic concentrations of the *E. cava* extract for adrenal cell proliferation, we treated human adrenal NCI-H295R cells with varying concentrations of the extract (0–100 μg/mL) for 24 h. Treatment with *E. cava* water extract alone did not interfere with NCI-H295R proliferation, and the average viability of cells exposed to the *E. cava* water extract was more than 90% for all concentrations evaluated (Figure 1A).

### *E. cava* Extract Inhibits FOR-Induced DHEA Production in NCI-H295R Cells

We further evaluated the effects of the *E. cava* extract on DHEA production in NCI-H295R cells as an established *in vitro* model of androgen generation in PCOS. To simulate the PCOS condition wherein androgen levels are elevated, the cells were exposed to FOR to induce androgen production. DHEA levels were analyzed in the conditioned culture medium. We observed that under FOR-stimulated conditions, exposure of the NCI-H295R cells to *E. cava* extract reduced DHEA synthesis in a dose-dependent manner (Figure 1B). Similar effects were observed for metformin and pioglitazone.

### *E. cava* Is Unlike Estrogen With Respect to Effects on *in vitro* Proliferation and pS2 mRNA Expression

To assess the estrogenic effects of *E. cava*, MCF-7 cell proliferation was examined after treatment with estrogen and *E. cava*. Treatment with estrogen increased MCF-7 cell proliferation compared to the control, but *E. cava* treatment did not lead to increased cell proliferation (Figure 1C). These data indicate that *E. cava* does not act in a manner similar to estrogen. We evaluated *pS2* gene expression via mRNA analysis; *pS2* is an estrogen-responsive gene. Similar to our observation for estrogen treatment, there was no increase in pS2 gene expression after *E. cava* treatment (Figure 1D). Therefore, we confirmed that *E. cava* does not act in a manner similar to estrogen and it is not estrogen responsive.

### Effect of *E. cava* Extract on Physiological Changes

**FIGURE 2:**
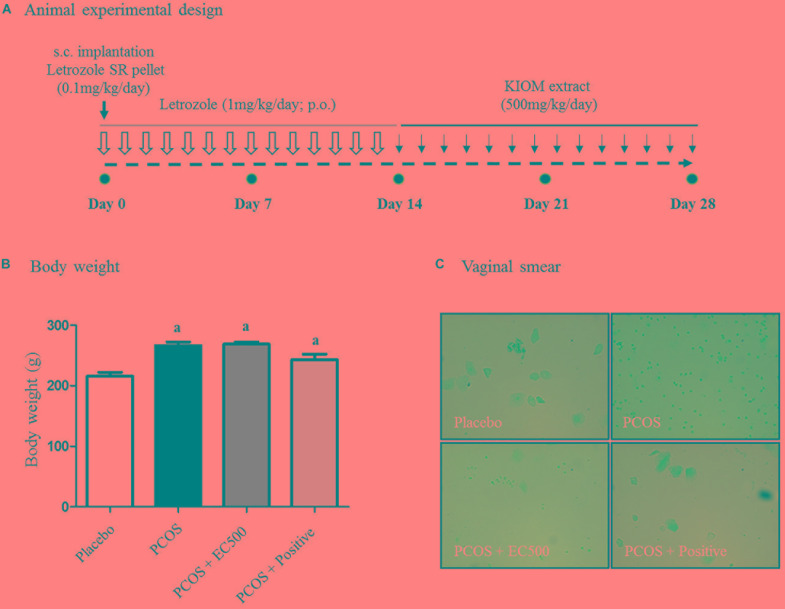
Effect of *E. cava* treatment on physiological changes. **(A)** For PCOS experiments, 6-week-old female rats were administered letrozole pellets (0.1 mg/kg/day). After 2 weeks, *E. cava* extract was administered via P.O. treatment every day for 2 weeks. Treatment of rats in the PCOS+positive groups with pellets was removed 14 d after insertion. **(B)** Body weights were measured on the last day; *^a^p* < 0.05 vs. placebo group. **(C)** Vaginal smears were conducted on the last day, and hematoxylin staining was performed. Magnification, 100 ×; section of rat ovary. EC, *E. cava* extract; PCOS, polycystic ovary syndrome.

To investigate the effects of the *E. cava* extract, rats with letrozole-induced PCOS were treated with *E. cava* extract (EC500; 500 mg/kg/day) for the final 2 weeks; induction was performed by insertion of letrozole pellets for 4 weeks (Figure 2A). When we monitored the body weights and estrus cycles, all rats with letrozole-induced PCOS exhibited increased body weights and a prolonged diestrus phase compared to the corresponding placebo control rats (Figure 2B). When rats with PCOS were first exposed to letrozole for 2 weeks (0.1 mg/kg/day), followed by 2 weeks of exposure to placebo pellets, the body weights of these PCOS + rats were not significantly different compared to those of the corresponding rats with PCOS exposed to letrozole for 4 weeks. To assess the letrozole-induced cycle arrest, we monitored the estrus cycle using vaginal smears. As expected, rats with PCOS exhibited a prolonged diestrus phase (Figure 2C). Microscopic observation of the vaginal smears revealed that the most common cell type was leukocytes in the rats with letrozole-induced PCOS. Interestingly, some EC500-treated rats with PCOS were observed to have regular cycles, similar to the placebo rats (Figure 2C). Our findings suggest that EC500 did not alter weight gain due to letrozole-induced PCOS but restored the regular cycle after letrozole-induced cycle arrest.

### Effect of *E. cava* on Plasma Hormonal Levels

**FIGURE 3:**
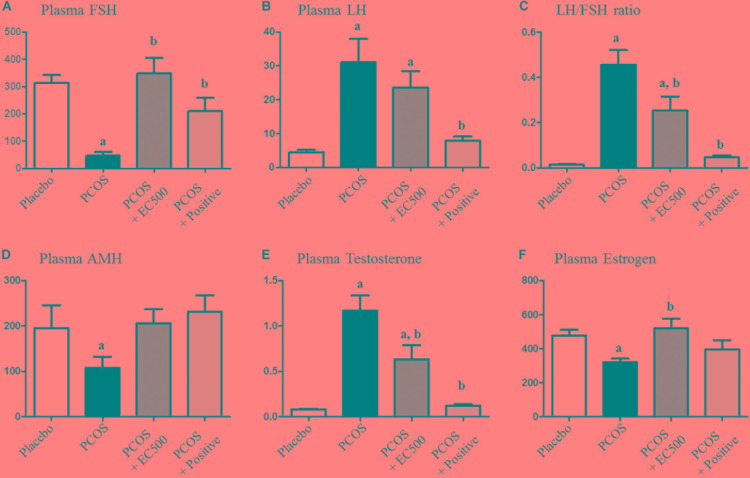
Effect of *E. cava* extract on plasma hormonal levels. For PCOS experiments, 6-week-old female rats were administered letrozole pellets (0.1 mg/kg/day). After 2 weeks, *E. cava* extract was administered via P.O. treatment every day for 2 weeks. Levels of plasma steroid hormones, such as **(A)** FSH, **(B)** LH, **(C)** LH/FSH ratio, **(D)** AMH, **(E)** testosterone, and **(F)** estrogen, were measured using a competitive enzyme-linked immunosorbent assay (ELISA) kit. All values represent the means ± SEMs. ^a^*p* < 0.05 vs. placebo group, ^b^*p* < 0.05 vs. PCOS group. EC, *E. cava* extract; PCOS, polycystic ovary syndrome.

The plasma FSH level (*p* < 0.05 vs. placebo) was significantly reduced in rats with letrozole-induced PCOS compared with placebo rats (Figure 3A). Similar to the PCOS + rats, the FSH levels in the PCOS + EC500-treated rats were similar to those observed in the placebo rats, and in turn, letrozole restored the reduced FSH levels (*p* < 0.05 vs. PCOS) (Figure 3A). In contrast, plasma LH responded robustly to letrozole treatment in PCOS rat models, irrespective of EC500 treatment (Figure 3B). As assessed by the LH/FSH ratio of plasma hormones, only rats in the EC500 treatment group exhibited a response in the LH/FSH ratio, unlike the corresponding rats with PCOS (Figure 3C). Moreover, plasma AMH levels (*p* < 0.05 vs. placebo) were significantly reduced in rats with letrozole-induced PCOS compared with the placebo rats (Figure 3D). As expected, the testosterone levels were significantly elevated in the rats with PCOS (Figure 3E). Interestingly, the letrozole-induced plasma testosterone levels were significantly decreased in the PCOS + EC500-treated rats (Figure 3E). As a positive control, PCOS + rats were not significantly different from the corresponding placebo rats. In contrast to testosterone levels, plasma estrogen levels were significantly decreased in the rats with PCOS, and the reduction in estrogen levels by letrozole was restored by EC500 treatment (Figure 3F).

### Effects of *E. cava* on mRNA Expression and Histological Changes During Folliculogenesis

**FIGURE 4:**
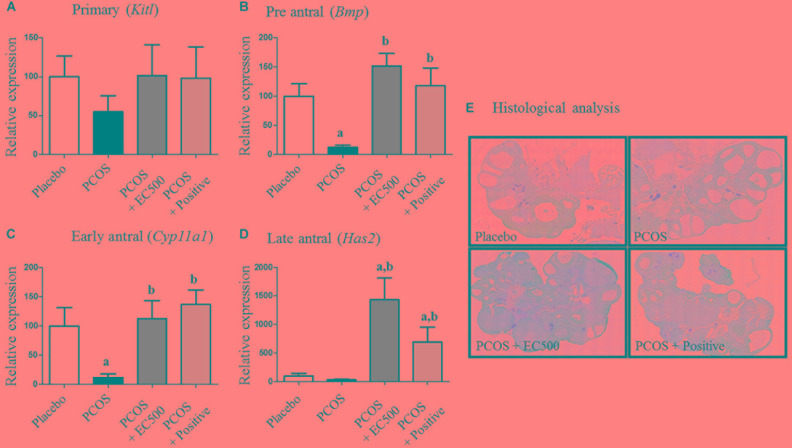
Effects of *E. cava* on the mRNA expression of folliculogenesis-related genes and sex steroid hormone receptors in the ovaries of rats. For PCOS experiments, 6-week-old female rats were administered letrozole pellets (0.1 mg/kg/day). After 2 weeks, *E. cava* extract was administered via P.O. treatment every day for 2 weeks. **(A)** *KitL*, **(B)** *Bmp*, **(C)** *Cyp11a1*, and **(D)** *Has2* mRNA expression was tested using RT-PCR in ovarian tissue from rats of each experimental group. Experiments were carried out in triplicate, and the data are expressed as the means ± SEM (*n* = 5–8). ^a^*p* < 0.05 vs. placebo group, ^b^*p* < 0.05 vs. PCOS group. **(E)** Histological analysis of an ovary and its sections stained with H&E. Magnification, 40×. EC, *E. cava* extract; PCOS, polycystic ovary syndrome.

Next, we investigated the effects of EC500 on the mRNA expression of ovarian *Kitl*, *Bmp*, *Cyp11a1*, and *Has2*, which are associated with follicle development; these genes can serve as biomarkers to diagnose the follicular stage of the ovarian cycle, such as the primary (*Kitl*), preantral (*Bmp*), early antral (*Cyp11a1*), and late antral (*Has2*) stages of the ovary (Epifano and Dean, 2002). In the present study, we measured *Kitl*, *Bmp*, *Cyp11a1*, and *Has2* gene expression to verify follicular development in rats with PCOS. The mRNA expression of *Kitl* was not significantly changed in any of the groups (Figure 4A); however, *Bmp*, *Cyp11a1*, and *Has2* expression was significantly increased in the positive group compared with the PCOS group. In addition, the mRNA levels of *Bmp*, *Cyp11a1*, and *Has2* were significantly elevated by EC500 treatment compared to the levels in the non-treated group (PCOS group). These results indicate that the expression of follicular markers, i.e., *Bmp* (Figure 4B), *Cyp11a1* (Figure 4C), and *Has2* (Figure 4D), was upregulated by EC500 treatment and may therefore be involved in improvement of follicular development in rats with letrozole-induced PCOS during the ovarian cycle (Epifano and Dean, 2002). In addition to changing the expression of follicular stage indicators, EC500 also induced follicle development in the presence of letrozole and was consistent in maintaining late antral follicles compared to PCOS + rats (Figure 4E). When the ovaries of rats with PCOS had numerous subcapsular cysts and thin granulosa cell layers, we observed numerous corpora lutea and antral follicles in the granulosa cell layer of PCOS + EC500-treated rats.

### Effects of *E. cava* on Gonadotropin-Related Genes

**FIGURE 5:**
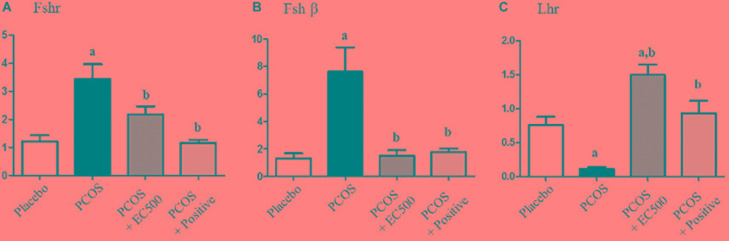
Effects of *E. cava* extract on gonadotropin-related genes. For PCOS experiments, 6-week-old female rats were administered letrozole pellets (0.1 mg/kg/day). After 2 weeks, *E. cava* extract was administered via P.O. treatment every day for 2 weeks. **(A)** *Fshr*, **(B)** *Fshβ*, and **(C)** *Lhr* mRNA levels were determined by quantitative RT-PCR. *Gapdh* mRNA was used as an internal control. Values represent the means ± SEM. ^a^*p* < 0.05 vs. placebo group, ^b^*p* < 0.05 vs. PCOS group. EC, *E. cava* extract; PCOS, polycystic ovary syndrome.

To estimate the transcript levels of gonadotropin-related genes, we performed real-time RT-PCR using specific primers for *Fshr*, *Fshβ*, and *Lhr*. Although the *Fshr* and *Fshβ* mRNA levels were significantly increased in rats with PCOS, these gonadotropin-related gene levels were significantly decreased in PCOS + EC500-treated rats compared to the corresponding rats with PCOS (Figures 5A,B). Similar to the placebo rats, the PCOS + rats also exhibited significantly decreased *Fshr* and *Fshβ* mRNA levels (Figures 5A,B). In contrast to *Fshr* mRNA, the plasma *Lhr* mRNA levels were significantly decreased in rats with PCOS, and the reduction in *Lhr* mRNA levels by letrozole was restored by EC500 treatment (Figure 5C).

### Effect of *E. cava* on Steroid Hormone-Related Genes

**FIGURE 6:**
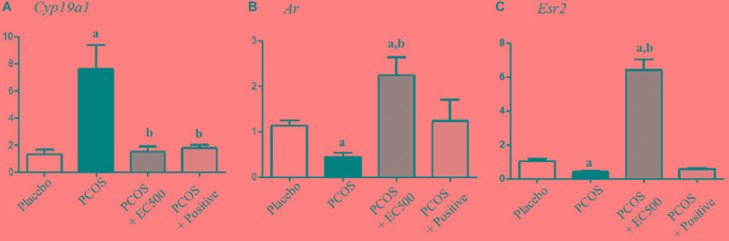
Effect of *E. cava* treatment on hormone receptor genes. For PCOS experiments, 6-week-old female rats were administered letrozole pellets (0.1 mg/kg/day). After 2 weeks, *E. cava* extract was administered via P.O. treatment every day for 2 weeks. **(A)** *Cyp19a1*, **(B)** *Ar*, and **(C)** *Esr2* mRNA levels were determined by quantitative RT-PCR. *Gapdh* mRNA was used as an internal control. Values represent the means ± SEM. ^a^*p* < 0.05 vs. placebo group, ^b^*p* < 0.05 vs. PCOS group. EC, *E. cava* extract; PCOS, polycystic ovary syndrome.

To assess the expression levels of mRNAs encoding enzymes involved in aromatase and sex steroid hormone receptors, we performed real-time RT-PCR. While significantly elevated in rats with PCOS, *Cyp19a1* mRNA levels were significantly decreased in PCOS + EC500-treated rats (Figure 6A). In addition to *Cyp19a1* mRNA, *Ar*, and *Esr2* receptor mRNAs were substantially and significantly suppressed in the PCOS ovaries (*p* < 0.05 vs. placebo). Furthermore, *Ar* and *Esr2* mRNA levels were significantly increased by *E. cava* extract treatment in rats with letrozole-induced PCOS (PCOS + EC500) compared to PCOS ovaries (Figures 6B,C).

### Immunohistochemical Localization and Expression of Aromatase, Androgen Receptor and Estrogen Receptor β

To assess the localization and expression of CYP19A1 (an aromatase), Ar and Erβ, immunohistochemistry was performed using specific antibodies. In both the placebo ovaries and

**FIGURE 7:**
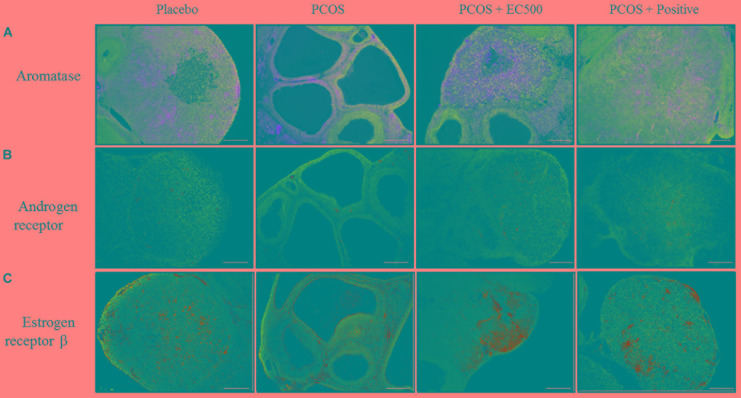
Effect of *E. cava* treatment on aromatase, androgen receptor, and estrogen receptor β localization. For PCOS experiments, 6-week-old female rats were administered letrozole pellets (0.1 mg/kg/day). After 2 weeks, *E. cava* extract was administered via P.O. treatment every day for 2 weeks. Immunohistochemistry was performed on ovarian sections using specific **(A)** aromatase (CYP19A1), **(B)** androgen receptor antibodies, and **(C)** estrogen receptor β antibodies. The secondary antibody was donkey and rabbit (scale bar = 200 μm). Magnification, 40 ×. EC, *E. cava* extract; PCOS, polycystic ovary syndrome.

PCOS + positive ovaries, ovarian aromatase, Ar and Erβ were detected within the granulosa cells of the follicle antrum (Figure 7). In rats with PCOS, most of the follicles were degenerated, and ovarian aromatase, Ar and Erβ were not detected in the granulosa cells of the degenerated follicles (Figure 7). More importantly, the EC500-treated

group of rats with letrozole-induced PCOS (PCOS + EC500) showed obvious changes in the developed follicles, and there was some indication of ovarian aromatase, Ar and Erβ expression in the granulosa cells of the follicle antrum; this effect was most notable in the PCOS + EC500 group. Interestingly, while the *Cyp19a1* mRNA levels significantly increased in the rats with PCOS, aromatase completely disappeared from the follicles. In parallel with the transcription levels, Ar and Erβ expression increased in the follicles of PCOS + EC500-treated rats compared to the corresponding rats with PCOS (Figure 7).

## Discussion

Polycystic ovary syndrome is a complex metabolic and endocrine disorder. This disorder is characterized by irregular ovulation, abdominal obesity and hyperandrogenism (Kafali et al., 2004). The estrus cycle is related to alterations in the circulating sex hormone, which controls ovarian function, including follicular maturation and hormonal imbalance (Sun et al., 2013). In our PCOS rat model, increased body weight was observed, and the estrus cycle was prolonged; however, the deficiencies were improved by treatment of the rats with PCOS with *E. cava* extract. In a previous study, rats with letrozole-induced PCOS also exhibited increased body weights, similar to those associated with PCOS in women (Kafali et al., 2004). Additionally, the rats with PCOS did not exhibit a regular estrus cycle and had a prolonged diestrus phase (Rajan et al., 2017). Using cell proliferation and expression of the *pS2* gene as estrogen-responsive markers, the effect of the *E. cava* extract was evaluated in MCF-7 cells. Cell proliferation and the mRNA levels of *pS2* were enhanced by 17β-estradiol; however, this enhancement was not observed in the *E. cava*-treated group. These results suggest that the *E. cava* extract does not have an estrogenic effect, and this result may not be associated with the estrous cycle in PCOS and/or normal female rats.

To validate the effect of the *E. cava* extract on hormonal changes, we examined the plasma hormone levels. Increased plasma levels of androgen and LH were the most consistent hormonal feature of rats with PCOS (Abbott et al., 2002), and low levels of progesterone and estradiol were also observed in rats with PCOS (Abbott et al., 2002). In this study, *E. cava* extract significantly upregulated the level of FSH or downregulated the levels of LH and LH/FSH in the serum compared to the levels in rats with PCOS. High levels of LH and increased LH:FSH ratios can serve as biomarkers to diagnose PCOS in women (Bednarska and Siejka, 2017). Additionally, the plasma testosterone levels were significantly decreased by *E. cava* extract treatment in rats with PCOS. In a previous study, excessive testosterone was shown to contribute to the pathogenesis of PCOS, and repression of these high levels of testosterone may have beneficial effects on disorders in PCOS (Abbott et al., 2002; Kafali et al., 2004). In contrast to testosterone levels, the serum levels of AMH and estrogen were decreased in letrozole-treated rats, and the reduction was correlated with early- or mid-follicular development and follicular morphology formation in the ovaries (Dewailly et al., 2016). Chemicals and hormones are the most effective remedies for the treatment of abnormal symptoms associated with female reproductive diseases; however, these substances are associated with several adverse effects, including uterine bleeding, hyperplasia and uncertain risks (Hidaka et al., 2013; Yang et al., 2018). However, recent studies have been conducted to mitigate the side effects of chemical drugs and hormone therapy via treatment with natural substances, and there has been an increase in the use of herbal formulas to treat symptoms in humans, including childbearing women (Cornish and Garbar, 2010; Hidaka et al., 2013; Lee et al., 2018). These results suggest that *E. cava* may be a potent therapeutic for the treatment of hormonal disturbances associated with PCOS and that this substance has potential applications as a functional food for women.

The number of developed follicles and the morphology of constitutive cells of the follicle in the ovary correspond to decreased levels of steroid hormones (Kafali et al., 2004; Baravalle et al., 2006). In our study, the components of the ovarian follicles were analyzed via potential markers, i.e., *Kitl*, *Bmp*, *Cyp11a1*, and *Has2*, and histological examination was performed. The mRNA levels of *Bmp*, *Cyp11a1*, and *Has2* were downregulated; however, this reduction was recovered by treatment with *E. cava* extract in the ovaries of PCOS rats. Histological analysis showed that the rats with PCOS also exhibited thin granulosa cell layers, decreased follicular development and numerous subcapsular cysts. Furthermore, the follicular cysts had a flattened epithelioid cell layers facing the antrum and thickened hyperplastic theca interna cells in the cyst walls. Previously, follicular dysfunction, such as atretic and large cysts with scant granulosa cells induced by letrozole, was also observed (Baravalle et al., 2006). In the present study, treatment with *E. cava* extract restored the components and morphologies of ovarian follicles to a normal range, suggesting that this extract is involved in the regulation of various factors associated with follicular development in the ovary.

In the present study, treatment with *E. cava* extract increased *Cyp19a1* mRNA expression in the ovaries of rats with PCOS. Previous studies have reported dysfunctional aromatase activity in PCOS women, and *Cyp19a1* plays a key role in the normal progression of the menstrual/estrous cycle in rats with PCOS (Kafali et al., 2004). In contrast, granulosa cells in polycystic ovaries have low aromatase activity, resulting in an imbalance in the production of estrogen and androgen (Chen et al., 2015). As shown here, consistent with the *Cyp19a1* mRNA levels, the immune-reactive aromatase of rats with PCOS was not detected in the granulosa cells or in degenerated follicles. Conversely, aromatase in the PCOS + EC500 rats was detected in the granulosa cells of the follicle antrum and exhibited considerable expression. In addition, the mRNA levels of *Ar* and *Esr2* were decreased; however, this downregulation was reversed by *E. cava* extract treatment in rats with PCOS in our study. The transcripts of *Ar* and *Esr2* have been reported to play a proliferative role during follicular development (Horie et al., 1992; Mosselman et al., 1996; Fitzpatrick et al., 1999), and increased *Ar* levels might result in enhancement of the proliferation and differentiation of granulosa cells (Hasegawa et al., 2017).

Moreover, the transcriptional levels of *Fshr*, *Fshβ*, and *Lhr* were altered in the ovaries of PCOS rats, and these levels were restored to the normal range by *E. cava* extract treatment. *Fshr*, synergistically with other stimulating factors such as androgens, regulates follicle growth moderately during the basal follicle growth phase (Dewailly et al., 2016). *Lhr* is also located on the surfaces of theca cells and granulosa cells, and *Lhr* transcript levels also influence ovulation, corpus luteum formation and the production of other steroids, i.e., estrogen, progesterone and androgen (Edson et al., 2009). Based on these results, we found that follicles in rats with PCOS were present in the primary or preantral stage. Interestingly, the *E. cava* extract improved the degeneration of follicle development.

The results presented in our report suggest that the *E. cava* extract has numerous beneficial effects on hormonal changes in rats with PCOS, and these changes significantly aid the recovery of the estrus cycle, the number of developed follicles and ovarian morphology in rats with PCOS. However, *E. cava* extract contains various compounds, and further studies on fractions form partial purification of the extract are required to identify the active pharmaceuticals among the phlorotannin components. These findings have uncovered a potential treatment for PCOS, therefore, we believe that the *E. cava* extract could be useful for managing PCOS in women.

## Author Contributions

HY, SYL, E-JH, and HL performed the research, analyzed the data, and wrote the manuscript. HY, SYL, SRL, HK, YL, and SK performed *in vivo* experiments and data analysis. B-JP performed *in vitro* experiments and data analysis. DS, CL, and HL prepared and supplied extract. All authors read and approved the final manuscript.

## Conflict of Interest Statement

The authors declare that the research was conducted in the absence of any commercial or financial relationships that could be construed as a potential conflict of interest.
