# Supplementary material for: Therapeutic Effect of Ecklonia cava Extract in Letrozole-Induced Polycystic Ovary Syndrome Rats
Source: Front Pharmacol. 2018 Nov 19;9:1325. doi: 10.3389/fphar.2018.01325 (PMC6262357; doi:10.3389/fphar.2018.01325)
Supplement: Supplementary file 1 [file Table_1.pdf]

Supplement Table S1. Quantification results for 5 metabolites in *E. cava* extract by MRM methods.

No.	Metabolites	Precursor ion (m/z)	Product ion (m/z)	µg of STD/ mg of sample
1	Eckol	371	263	1.01246µg/mg
2	Dieckol	741	261	4.6478µg/mg
3	6,6’-bieckol	741	477	4.6444µg/mg
4	8,8’-bieckol	741	723	1.1841µg/mg
5	Phloroglucinol	125	57	0.185µg/mg
