# Supplementary figures and images for: Therapeutic Effect of Ecklonia cava Extract in Letrozole-Induced Polycystic Ovary Syndrome Rats
Source: Front Pharmacol. 2018 Nov 19;9:1325. doi: 10.3389/fphar.2018.01325 (PMC6262357; doi:10.3389/fphar.2018.01325)

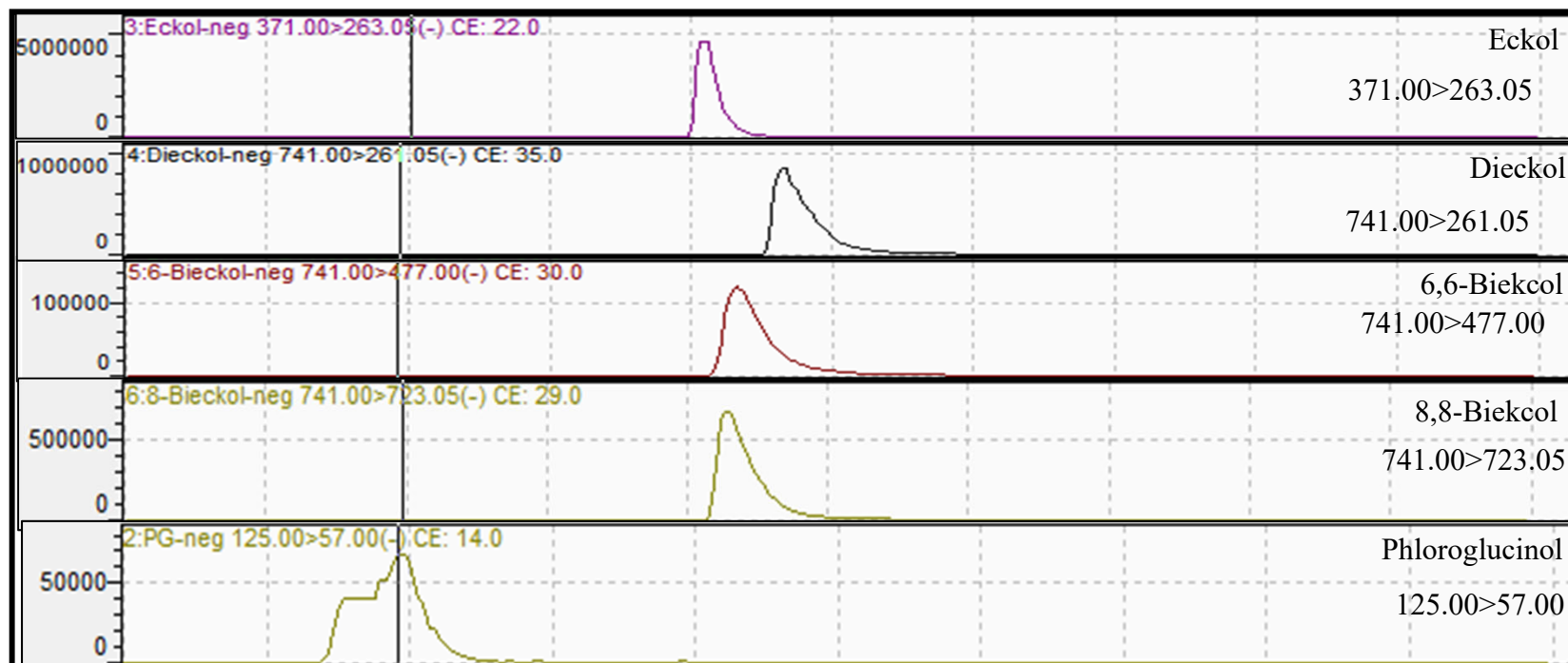

**Supplement Figure S1.** LC-MS chromatograms of 5 reference compounds analyzed in MRM modes.

Supplement: Supplementary file 2 [file Image_1.pdf]
